# Comparative Study of Quality Characteristics of Korean Soy Sauce Made with Soybeans Germinated Under Dark and Light Conditions

**DOI:** 10.3390/ijms12118105

**Published:** 2011-11-17

**Authors:** Ung-Kyu Choi, Yeon-Shin Jeong, O-Jun Kwon, Jong-Dae Park, Young-Chan Kim

**Affiliations:** 1 Department of Food Science and Technology, Chungju National University, Chungbuk 368-701, Korea; E-Mail: ukchoi@cjnu.ac.kr; 2 Institute of Agricultural Science and Technology, Kyungpook National University, Daegu 702-701, Korea; E-Mail: soyabean@knu.ac.kr; 3 Gyeongbuk Regional Innovation Agency, Gyeongsan 712, Korea; E-Mail: ojkwon@gbria.or.kr; 4 Korea Food Research Institute, Seongnam 463, Korea; E-Mail: jdpark@kfri.re.kr

**Keywords:** germination, light condition, color, Korean soy sauce, isoflavone

## Abstract

This study was conducted to evaluate the effects of germinating soybeans under dark and light conditions on the quality characteristics of Korean soy sauce made with germinated soybeans. The germination rate of soybeans germinated under dark conditions (GSD) was higher than that of soybeans germinated under light conditions (GSL), whereas the lengths of sprouts and relative weights of GSL did not differ from those of GSD. The *L*, *a*, *b*, and Δ*T* values of GSL were significantly lower than GSD. The color of GSD remained yellow, while GSL changed to a green color due to photosynthesis by chlorophyll. The total amino acid contents in soy sauce fermented with soybeans germinated under dark conditions (SSGD) and soy sauce fermented with soybeans germinated under light conditions (SSGL) were lower than in soy sauce fermented with non-germinated soybeans (SNGS). The levels of isoflavone content in SSGD and SSGL were significantly increased compared to the SNGS. In conclusion, the germination of soybeans under dark and light conditions is not only an increasing organoleptic preference, but also has implications for the health benefits of Korean soy sauce.

## 1. Introduction

Soybean, a major source of vegetable protein and oil, provides various nutritional benefits to a large part of the world’s population, especially in Asian countries. It is a nutrition-rich foodstuff which supplies macronutrients such as proteins, fatty acids, fiber, and carbohydrates. It also provides diverse functional compounds such as isoflavones, soy saponins, and vitamin E, which have been known to exhibit various biological activities [1].

Isoflavones, one of the most abundant functional ingredients of soybean, are selective estrogen receptor modulators or biochemical compounds that are able to agonize or antagonize estrogen receptors [2]. The intake of soybean isoflavones has been proved to be associated with a decreased risk of some diseases [3]. Many studies have demonstrated that isoflavones are not only effective against cancer [4,5] but also reduce fat deposition *in vivo* and *in vitro* [6,7]. There is solid evidence supporting the idea that soy isoflavone may produce some degree of neuroprotection [8,9].

Because germination may cause changes in the nutrients and functional substances of seeds through aerobic respiration and biochemical metabolism [10], germination processes for soybeans have been developed to overcome the disadvantages of seeds used in food products and make sprouts safe for human consumption. Such disadvantages include undesirable flavor and odor due to lipoxygenase activity, and the presence of antinutritional factors such as trypsin inhibitors, phytates, and flatulent [11,12]. The amount of total isoflavones has been shown to increase within a short germination period (6–24 h), and gradually decrease thereafter [13]. It was found that daidzein, genistein, and their respective conjugates are the major soluble isoflavonoids in seedlings, roots, hypocotyls, and cotyledons after germination [14]. However, information on the effect of soybean germination on isoflavone content and composition is still limited [15].

Traditional Korean fermentation products such as soy sauce (*kanjang*), soybean paste (*doenjang*), and Korean fermented red pepper paste (*gochujang*) are produced through the fermentation of soybeans by naturally occurring bacteria and fungi, and have been consumed for centuries as protein sources and flavoring ingredients in Korea. Among them, Korean soy sauce (KSS) is traditionally prepared as a liquid seasoning by mixing and fermenting moldy cooked soybeans (*meju*) with brine in a container such as a porcelain pot [16]. Soy sauce contains certain bioactive components in addition to its taste and aromatic compounds, and various biological activities of soy sauce have been reported. Such activities include anticarcinogenic properties [17], antimicrobial activity [18], antioxidant activity [19], anti-platelet aggregation activity [20], and angiotensin I-converting enzyme inhibition activity [21].

We previously reported that isoflavone contents were significantly increased in *meju* made with germinated soybeans under both dark conditions [22] and light conditions [23], as compared to *meju* made with non-germinated soybeans. The present investigation was conducted to determine the comparative effects of germinating soybeans under dark and light conditions on the quality characteristics of *kanjang*, a Korean soy sauce (KSS), fermented with germinated legume including the content of taste components and isoflavones.

## 2. Results

### 2.1. Germination Characteristics of the Soybeans Sprouted Under Dark and Light Conditions

**Figure 1:**
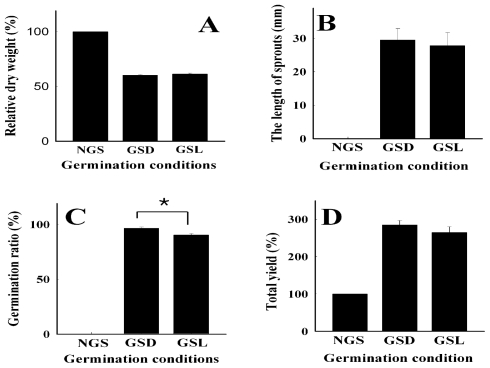
Germination characteristics of soybeans. (**A**) Relative dry weight; (**B**) The length of sprouts; (**C**) Germination ratio; (**D**) Total yield. Each value was determined after 48 h of germination at 20 °C und der dark or light conditions. Results are representative of 20 independent experiments. *p < 0.05. NGS, Non-germinated soybeans; GSD, Germinated soybeans under dark conditions; GSL, Germinated soybeans under light conditions.

The relative dry weights of the soybeans germinated for 48 h under dark and light conditions were 60.1% ± 0.9% and 61.4% ± 0.7%, respectively, as shown in Figure 1A. The relative dry weights of soybeans germinated under dark conditions (GSD) and under light condition (GSL) were not significantly different at 48 h after germination. The root lengths of the soybeans germinated under dark and light conditions are shown in Figure 1B. At 48 h after germination, the root lengths of GSD and GSL were 29.5 ± 3.4 and 27.8 ± 3.9 mm, respectively. The difference in growth rate between GSD and GSL was insignificant at the 95% level, although a slightly more rapid growth was observed with dark germination. The germination rates of soybeans germinated under dark and light conditions for 48 h were measured by adding water for 2 h after the soybeans had soaked for 4 h, and plating them in a constant-temperature room controlled at 25 °C. At 48 h after plating, the germination rate of GSD (96.5% ± 1.1%) was higher than that of GSL (90.4% ± 1.1%) (Figure 1C). The relative weight of the soybeans after soaking was 176 ± 8% as compared to the dried soybeans. Under dark conditions, the total yield of soybeans reached a level of 285% ± 12% after 48 h of germination, as described in Figure 1D. Although, the total yield of GSL (265% ± 15%) was slightly lower than the yield of GSD, light exposure did not have a significant effect on the total weight of soybean seeds at 48 h after germination.

### 2.2. Changes in Soybean Color by Germination Under Dark and Light Conditions

**Figure 2:**
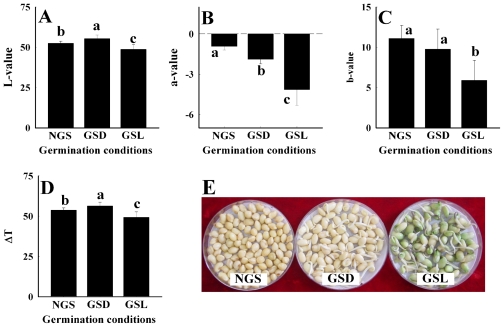
Changes in colors of germinated soybeans under dark and d light conditions. (**A**) *L*-value; (**B**) *a*-value; (**C**) *b*-value; (**D**) Δ*T* value; (**E**) Appearance of germinated soybeans under dark and light conditions. Each value was determined after 48 h germination at 20 °C under dark and light conditions. Results are representative of 20 independent experiments. In a column, mean values followed by the same letter are not significantly different at the 5% level. NGS, Non-germinated soybeans; GSD, Germinated soybeans under dark conditions; GSL, Germinated soybeans under light conditions.

A comparison between the color of soybeans germinated under dark and light conditions is shown in Figure 2. It was found that the *L*, *a*, *b*, and Δ*T* values of GSL were significantly lower than those of GSD. The active changes in *L*-value were identified by germination both under dark and light conditions. The *L*-value of GSL was lower than that of GSD (Figure 2A). The *a*-value of both GSD and GSL definitely differed from that of non-germinated soybeans (NGS) (Figure 2B). The *b*-value of GSL was significantly lower than that of GSD, which is not significantly different from the *b*-value of NGS (Figure 2C). The changing patterns of Δ*T* by germination under different conditions were very similar with those of the *L*-value (Figure 2D). Figure 2E shows the appearance of the soybeans germinated with and without light exposure. The color of soybeans germinated under dark conditions was yellow, while soybeans exposed to light conditions changed to a green color due to the photosynthesis by chlorophyll.

### 2.3. Comparison of Free Amino Acid Contents in KSS Fermented with Soybeans Germinated under Dark and Light Conditions

The free amino acid contents in KSS according to the conditions of germination of the raw soybeans are given in Table 1. The total amino acid content in soy sauce fermented with soybeans germinated under dark conditions (SSGD) (2411.2 ± 89.5 mg%) and soy sauce fermented with soybeans germinated under light conditions (SSGL) (2451.6 ± 171.5 mg%) was lower that of soy sauce fermented with non-germinated soybeans (SNGS) (2746.1 ± 141.1 mg%). There were no significant differences in total amino acid content between SSGD and SSGL. The contents of savory and bitter tasting compounds in SSGD and SSGL were lower than in SNGS. Among the sweet tasting compound, threonine and lysine in SSGD and SSGL was lower than those in SNGS. Further the content of proline, valine, tyrosin, and phenylalanine in SSGD and SSGL was lower than those in SNGS. In this study, the levels of most amino acids except alanine, cystine, methionine, histidine, and arginine in both SSGD and SSGL were lower than in SNGS. The content of glutamic acid was highest in all tested KSS, followed by contents of lysine, leucine, and serine. The relative content of glutamic acid, which is the most abundant amino acid in soybean and soy sauce and accounts for 18.6~18.7% of total amino acids, did not differ among the samples. The content of essential amino acids in both SSGL and SSGD was lower than in SNG, and its percentage in SNGS, SSGD, and SSGL was 42.6%, 42.2%, and 41.8%, respectively.

### 2.4. Comparison of Isoflavone Contents in KSS Fermented with Soybeans Germinated Under Dark and Light Conditions

The isoflavone contents of SNGS, SSGD, and SSGL are given in Table 2. The levels of isoflavones in SSGD (621.6 ± 47.9 μg/g) and SSGL (605.9 ± 35.8 μg/g) were significantly increased as compared to their levels in SNGS (447.4 ± 28.49 μg/g). The level of genistin was the highest in all tested KSS. There were no significant differences observed between SSGD and SSGL. Among the individual types of isoflavones, the contents of daidzein, genistein, daidzin, genistin, and glacitein in SSGD and SSGL were higher than in SNGS. In contrast, glacitin content was not different among the tested KSS. Light exposure of the soybeans during germination had a slight impact on the isoflavone levels in this study. Although, significant effect from light exposure was not observed on the isoflavone levels of soybeans after 48 h of germination, the total isoflavone content in SSGD was higher than those in SSLG.

### 2.5. Sensory Evaluation of KSS Fermented with Soybeans Germinated Under Dark and Light Conditions

The sensory evaluation results of the KSS made with soybeans germinated under dark and light conditions are shown in Table 3. Although, the color, odor, sweet taste, sour taste, and overall acceptability of SSGD and SSGL were slightly higher than for SNGS, these parameters did not show significant differences among the tested KSS. The salty taste of SSGD and SSGL were slightly lower than that of SNGS. No significant increase or decrease of KSS taste parameters was observed due to the light exposure of raw soybeans during germination.

## 3. Experimental Section

### 3.1. Materials

The soybeans (Glycine max: Eunhakong) used in this experiment were purchased from Soyventure Co. Ltd. (Daegu, Korea) and were harvested in 2009. The proximate composition of soybean was: moisture (8.1%), ash (4.9%), crude protein (39.2%), crude fat (19.4%), crude fiber (5.7%), and nitrogen free extract (22.7%).

### 3.2. Germination of Soybeans Under Dark and Light Conditions

The washed soybean seeds were soaked in water at 20 °C at for 4 h and then transferred into a culture container (25 cm × 25 cm × 30 cm), and the culture containers were placed in thermostat dark and light houses (1000 lux). The soaked soybean seeds were cultivated for 48 h under a top-irrigation system commercially used for soybean sprout production. Irrigation was provided for 3 min every 2 h with underground water. The average temperature range inside the culture house during the experiments was 20–25 °C.

### 3.3. Preparation of Korean Soy Sauce

**Figure 3:**
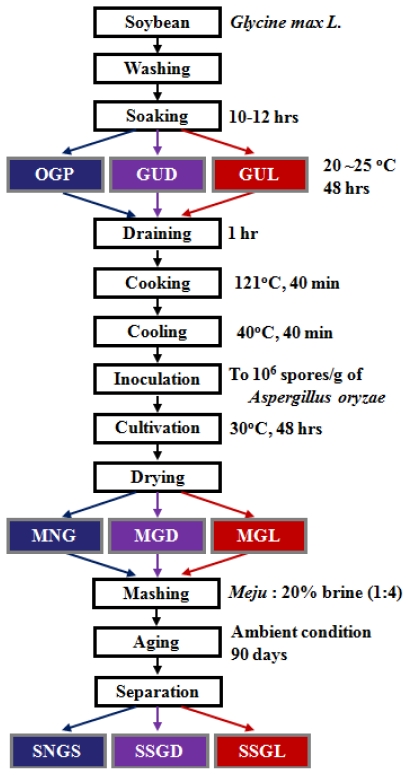
Protocol for the preparation of Korean soy sauce made with soybeans germinated under dark and light conditions. OGP, Omission of germination process; GUD, Germination under dark conditions; GUL, Germination under light conditions (1000 lux); MNG, *Meju* fermented with non-germinated soybeans; MGD, *Meju* fermented with soybeans germinated under dark conditions; MGL, *Meju* fermented with soybeans germinated under light conditions; SNGS, soy sauce fermented with non-germinated soybeans; SSGD, soy sauce fermented with soybeans germinated under dark conditions; SSGL, soy sauce fermented with soybeans germinated under light conditions.

The method used for producing the soy sauce made with germinated soybeans is described in Figure 3. First, the germinated soybeans were drained for 1 h, placed in an Erlenmeyer-flask, steamed for 40 min at 121 °C, and cooled to 40 °C. Then, *Aspergillus oryzae*, isolated and identified from a commercial *meju*, was inoculated to the steamed and germinated soybeans at 10^6^ spores/g. The whole soybean *meju* made with germinated soybeans was prepared by culturing the inoculated steamed soybeans at 30 °C for 72 h and drying with a hot air dryer (SGO-100, Sunggun, Korea) for 2 days. Finally, 200 g of dried whole soybean *meju* mixed with 21% salt water was matured for 3 months.

### 3.4. Measurement of Germination Characteristics

Total yield was expressed as the percentage of harvested sprout weight relative to the dry seed weight, as described in Equation (1). The germination ratio was expressed as the percentage of the number of germinated soybeans relative to the number of total soybeans, as shown in Equation (2). Twenty germinated soybeans (Eunhakong) were randomly selected after 48 h of germination, and sprout lengths were measured with a vernier caliper (5/100 m, Mitutoyo, Gawasaki, Japan).

(1) Total yield (%)=[Wt. of germinated soybeans (g)/Wt. of dried soybeans (g)]×100

(2) Germination ratio (%)=[No. of germinated soybeans (EA)/Total soybeans (EA)]×100

### 3.5. Color Measurement

The color measurements of soybeans germinated under dark and light conditions were made with a chromometer (Chromometer CR 300, Minolta, Japan) and were calibrated with a white standard plate (*L* = 97.51, *a* = −0.18, *b* = +1.67).

### 3.6. Measurement of Free Amino Acids

The desalted sample was homogenized and extracted with 1% picric acid, and then passed through a Dowex 2 × 8 (Cl-form, 100–200 mesh) column filled with sodium citrate buffer (pH 2.2) to remove and evaporate the picric acid. The extract was filtered by a membrane filter (0.45 μm) and injected into an automatic amino acid analyzer (Biochrom 20 amino acid analyzer, Uppsala, Sweden) and quantified. The analysis conditions were as follows: buffer flow rate: 20 mL/h; ninhydrin flow rate: 20 mL/h; temperature gradient: 35, 74, 80, and 37 °C; wavelength decreased from 570 nm to 440 nm; column length: 46 mm × 250 mm; and injection volume: 20 μL.

### 3.7. Quantification of Isoflavones

The analysis of isoflavones was performed using a previously described modified method [24,25]. A 20 μL aliquot of filtrate was injected into a HPLC equipped with a Bondapack C18 column, after the system had been equilibrated at ambient temperature, and the UV detector had been stabilized with the mobile phase (methanol: 1 mM ammonium acetate, 6:4) at a flow rate of 1 mL/min for 30 min. The effluent was detected at 254 nm and the chromatogram was recorded for 20 min. The isoflavones were identified by the retention times of the added standards, and their contents were calculated by comparing their peak areas with those of the standards.

### 3.8. Sensory Evaluation

To evaluate the quality of the produced Korean soy sauces, 17 well-trained panelists performed a 7-point sensory test for external appearance, odor, taste, and overall acceptance. The panel members were aware of the experiment’s objective and were trained to evaluate the taste of the Korean soy sauce. The grading was on a scale of 7 (best) to 1 (worst), and an average score was 4. In each sensory test, 3 kinds of Korean soy sauce samples were placed into a bowl and given to each panel member; a randomly assigned 3-digit number was given to each sample. Analysis of variance (ANOVA), Student’s *t*-test and duncan’s multiple range test were performed using the Minitab program.

### 3.9. Statistical Analysis

The data were statistically analyzed by using the Student’s *t*-test, and analysis of variance for individual parameters was performed by Duncan’s test on the basis of mean values to determine significance at *p* < 0.05.

## 4. Discussion

Germination is the fastest growing period of plant life and is known to increase essential nutrient levels, bioavailability, and palatability of soybeans [26,27]. During this period, anti-nutritional factors and “beanny” flavors resulting from trypsin inhibitors, lipoxygenase, and hemagglutinins are also reduced. Therefore, greater utilization of germinated soybeans in the preparation of human and animal foods would be very beneficial. This study was undertaken to determine the effects of germinating soybeans under dark and light conditions on the quality characteristics of KSS, including the contents of amino acids and isoflavones.

The color of the soybeans dramatically changed during germination in our study, and the color change of GSL was more visible than that of GSD. However, our previous study reported that the color of *meju* fermented with soybeans germinated under light and dark conditions was not significantly different [23]. Furthermore, the *L* and *b*-values of the *meju* did not show large differences with the germination degree of raw soybeans under dark conditions, while the *a*-value of *meju* was slightly increased as the germination degree of raw soybeans increased [22]. It was presumed that this change was due to the chlorophyll generated during the germination under light conditions being destroyed through the boiling process.

In our study, the germination rate of GSD was higher than that of GSL at 48 h of germination. Choi *et al*. was reported that the germination rate was increased rapidly at 3 h of germination and the germination ratio was reached to the level of 99.4% at 60 h of germination [10]. Further the sprouting rate of soybean under dark condition was higher than that under light condition [23].

Compounds responsible for food taste commonly having low molecular weights and non-volatile properties. Free amino acids are the low molecular compounds that have a strong impact on the taste of soy sauce in the presence of sodium salt. In this study, the total amino acid content of KSS was significantly influenced by the germination of raw soybeans. However, exposure to light did not have an effect on the amino acid content of KSS. We previously examined the free amino acid content of *meju*, a Korean soybean fermentation starter, made by soybeans germinated under dark and light conditions, and no significant differences were observed in the total free amino acid contents of whole soybean *meju* as a result of light exposure during germination of the raw soybeans [23]. Although, *meju* is a main ingredient used to make KSS, due to the large differences in fermentation conditions employed, a direct comparison of results from two different studies is not appropriate. Among free amino acids, threonine, serine, glycine, alanine, and lysine present the sweet taste. The savory taste of soy sauce mainly results from by glutamic acid, aspartic acid and cysteine. Methionine, isoleucine, and the leucine present the bitter taste [28]. In this study, the germination status of soybeans had a significant effect for decreasing savory and bitter tasting compounds. Further, the level of some amino acid from sweet and others tastes were also significantly decreased in SSGD and SSGL.

The isoflavone contents in soybeans are affected not only by the soybean variety, but also by environmental changes in parameters such as temperature, sunshine duration, and precipitation. The isoflavone content in soybeans is in the order of genistein > daidzein > glycitein, and remained in that order as germination progressed. The total isoflavone content in soybeans rapidly increased with the beginning of the germination process, and reached a maximum level after 24 h of germination, when the hypocotyl length was ~0.5–2.5 mm in length, and decreased thereafter [29]. Consequently, the isoflavone content of soybeans germinated for 60 h was the same as that of non-germinated soybeans. Thus, controlled germination can be used to enhance isoflavone content in soybeans. It was reported that isoflavone content in soymilk made with germinated soybean was significantly increased, and the highest evaluated preference was for soymilk produced with beans that had germinated for 12 h [30]. We previously reported a similar result, in that isoflavone content in *meju* made with soybeans germinated for 24~48 was significantly increased compared to isoflavone content in *meju* made with non-germinated soybeans. Light exposure during germination resulted in increased levels of isoflavones [31]. However, this effect of light on isoflavones could not be confirmed by another study which concluded that light does not have a significant effect on the content of isoflavones during germination [32]. It has also been reported that light has varying effects on the concentrations of some minerals in soybean sprouts [33,34]. Additional information concerning the effects of light on other nutrients, such as tocopherols, phytosterols, and some of the macronutrients during germination, is still absent.

Sensory characteristics of KSS were not decreased by the germination of raw soybeans under either dark or light conditions, implying that the preference of KSS was not significantly changed by the germination conditions of the raw soybeans. This result was similar to our previous data in which odor, sweet taste, savory taste, bitter taste, and overall acceptability did not show significant differences among the cheonggugjangs made with different germination times [22]. Consequently, from the commercial point of view, germination of raw soybeans not only maintains the consumer’s gustative preference for the KSS, but also increases the isoflavone content in both SSGD and SSGL.

In conclusion, we found that during germination, both the factors of darkness and light potentially influence the quality characteristics of KSS. Germination of soybeans under dark or light conditions is not only increasingly an organoleptic preference, but also provides health benefits for the consumption of various soy food preparations. Thus, from the above results, it can be concluded that these factors are valuable for determining the nutritional value of soybean fermented products. Further studies are required to reveal macro and micronutrient levels in the KSS fermented with soybeans germinated under dark or light conditions.

## Figures and Tables

**Table 1:** Add Amino acid contents of Korean soy sauce fermented with germinated soybean under dark and light conditions. (Unit: mg%).

Amino acid		Germination condition of soybean		

		SNGS	SSGD	SSGL
Sweet taste	Thr	128.6 ± 6.6 ^a^	112.1 ± 3.2 ^b^	113.1 ± 6.8 ^b^
	Ser	190.0 ± 9.8 ^a^	167.7 ± 7.5 ^b^	171.5 ± 13.2 ^ab^
	Gly	88.7 ± 4.5 ^a^	82.2 ± 9.7 ^a^	87.7 ± 12.3 ^a^
	Ala	162.8 ± 8.4 ^a^	150.2 ± 17.0 ^a^	160.2 ± 22.0 ^a^
	Lys	301.8 ± 15.6 ^a^	257.5 ± 8.0 ^b^	254.7 ± 11.1 ^b^
	Subtotal	871.9 ± 44.8 ^a^	769.8 ± 34.5 ^b^	787.1 ± 60.9 ^ab^

Savory taste	Asp	183.9 ± 9.5 ^a^	156.1 ± 6.1 ^b^	153.3 ± 6.5 ^b^
	Glu	513.8 ± 26.7 ^a^	449.2 ± 15.2 ^b^	456.0 ± 30.5 ^b^
	Cys	15.0 ± 0.7 ^a^	14.5 ± 2.2 ^a^	15.5 ± 2.6 ^a^
	Subtotal	712.7 ± 36.8 ^a^	619.7 ± 17.0 ^b^	624.8 ± 36.9 ^b^

Bitter taste	Met	53.9 ± 2.7 ^a^	51.4 ± 8.0 ^a^	55.9 ± 9.7 ^a^
	Ile	140.7 ± 7.2 ^a^	123.1 ± 4.1 ^b^	124.8 ± 8.2 ^b^
	Leu	271.8 ± 14.0 ^a^	234.3 ± 5.7 ^b^	234.1 ± 11.7 ^b^
	Subtotal	466.4 ± 23.9 ^a^	408.8 ± 14.2 ^b^	414.8 ± 28.0 ^b^

Others	Pro	158.5 ± 8.1 ^a^	138.6 ± 4.4 ^b^	140.2 ± 9.0 ^b^
	Val	140.0 ± 7.2 ^a^	122.9 ± 4.5 ^b^	124.9 ± 8.6 ^b^
	Tyr	53.1 ± 2.6 ^a^	45.5 ± 1.5 ^b^	44.6 ± 1.8 ^b^
	Phe	133.9 ± 6.9 ^a^	115.9 ± 2.8 ^b^	116.0 ± 6.1 ^b^
	His	50.9 ± 2.5 ^a^	46.4 ± 4.1 ^a^	48.6 ± 5.6 ^a^
	Arg	158.6 ± 8.1 ^a^	143.7 ± 12.1 ^a^	150.7 ± 17.0 ^a^
	Subtotal	695.1 ± 35.5 ^a^	613.0 ± 25.6 ^b^	624.9 ± 46.4 ^ab^

Glu/TA (%)		0.187	0.186	0.186
EAA		1170 ± 60.2 ^a^	1017.2 ± 26.6 ^b^	1023.4 ± 58.6 ^b^
EAA/TA (%)		42.6	42.2	41.8
Total		2746.1 ± 141.1 ^a^	2411.2 ± 89.5 ^b^	2451.6 ± 171.5 ^b^

In a column, mean values followed by the same letter are not significantly different at the 5% level. Results are representative of 3 independent experiments. SNGS, soy sauce fermented with non-germinated soybeans; SSGD, soy sauce fermented with soybeans germinated under dark conditions; SSGL, soy sauce fermented with soybeans germinated under light conditions; EAA, Essential amino acid; TA, total amino acids.

**Table 2:** Isoflavone contents of Korean soy sauce fermented with germinated soybeans under dark and light conditions. (Unit: mg%).

Isoflavone	Germination condition of soybean		

	SNGS	SSGD	SSGL
Daidzein	36.3 ± 2.2 ^b^	47.9 ± 6.4 ^a^	55.6 ± 6.6 ^a^
Genistein	26.2 ± 2.7 ^b^	56.8 ± 4.4 ^a^	54.7 ± 6.7 ^a^
Daidzin	36.7 ± 3.6 ^b^	54.8 ± 4.4 ^a^	46.6 ± 5.3 ^a^
Genistin	302.4 ± 19.6 ^b^	407.9 ± 27.5 ^a^	389.0 ± 24.6 ^a^
Subtotal	401.7 ± 26.8 ^b^	567.4 ± 41.0 ^a^	545.9 ± 28.2 ^a^

Glacitein	7.6 ± 0.9 ^b^	15.9 ± 4.4 ^a^	21.1 ± 5.0 ^a^
Glacitin	38.2 ± 2.3 ^a^	38.3 ± 2.5 ^a^	39.0 ± 2.9 ^a^
Subtotal	45.8 ± 1.7 ^b^	54.2 ± 6.9 ^a^	60.0 ± 7.8 ^a^

Total	447.4 ± 28.4 ^b^	621.6 ± 47.9 ^a^	605.9 ± 35.8 ^a^

In a column, mean values followed by the same letter are not significantly different at the 5% level. Results are representative of 3 independent experiments. SNGS, soy sauce fermented with non-germinated soybeans; SSGD, soy sauce fermented with soybeans germinated under dark conditions; SSGL, soy sauce fermented with soybeans germinated under light conditions.

**Table 3:** Add Sensory evaluation score of Korean soy sauce fermented with germinated soybeans under dark and light conditions.

Sensory characteristics	Germination condition of soybean		

	SNGS	SSGD	SSGL
Color	4.2 ± 0.8 ^a^	4.2 ± 1.2 ^a^	4.4 ± 1.4 ^a^
Odor	4.4 ± 1.2 ^a^	4.6 ± 1.3 ^a^	4.6 ± 1.2 ^a^
Sweet taste	4.1 ± 1.1 ^a^	4.5 ± 1.2 ^a^	4.7 ± 1.4 ^a^
Saulty taste	4.8 ± 1.3 ^a^	4.6 ± 1.2 ^a^	4.5 ± 1.1 ^a^
Bitter taste	4.5 ± 1.0 ^a^	4.4 ± 1.3 ^a^	4.7 ± 1.3 ^a^
Sour taste	4.0 ± 0.9 ^a^	4.5 ± 1.3 ^a^	4.6 ± 1.1 ^a^
Savor taste	4.3 ± 0.8 ^a^	4.3 ± 1.0 ^a^	4.4 ± 1.3 ^a^
Overall accepability	4.4 ± 1.1 ^a^	4.5 ± 1.2 ^a^	4.6 ± 1.0 ^a^

Each value indicates the average of sensory scores, ranging from 1 (dislike extremely) to 7 (like extremely), recorded by 17 well-trained panelists. In a column, mean values followed by the same letter are not significantly different at the 5% level. Results are representative of 3 independent experiments. SNGS, soy sauce fermented with non-germinated soybeans; SSGD, soy sauce fermented with soybeans germinated under dark conditions; SSGL, soy sauce fermented with soybeans germinated under light conditions.

## Abbreviations

OGP: Omission of germination process
GUD: Germination under dark conditions
GUL: Germination under light conditions (1000 lux)
MNG: *Meju* fermented with non-germinated soybeans
MGD: *Meju* fermented with soybeans germinated under dark conditions
MGL: *Meju* fermented with soybeans germinated under light conditions
SNGS: Soy sauce fermented with non-germinated soybeans
SSGD: Soy sauce fermented with soybeans germinated under dark conditions
SSGL: Soy sauce fermented with soybeans germinated under light conditions
